# Anti-apoptotic effects of osteopontin through the up-regulation of Mcl-1 in gastrointestinal stromal tumors

**DOI:** 10.1186/1477-7819-12-189

**Published:** 2014-06-20

**Authors:** Kai-Hsi Hsu, Hung-Wen Tsai, Pin-Wen Lin, Yun-Shang Hsu, Pei-Jung Lu, Yan-Shen Shan

**Affiliations:** 1 Department of Surgery, Tainan Hospital, Ministry of Health and Welfare, Tainan 70428, Taiwan; 2 Department of Surgery, National Cheng Kung University, College of Medicine, Tainan 70428, Taiwan; 3 Institute of Clinical Medicine, National Cheng Kung University, College of Medicine, 138, Sheng-Li Road, Tainan 70428, Taiwan; 4 Department of Pathology, National Cheng Kung University, College of Medicine, Tainan 70428, Taiwan; 5 Department of Biotechnology, Southern Taiwan University of Technology, Tainan 70428, Taiwan

**Keywords:** Apoptosis, Gastrointestinal stromal tumor, Mcl-1, Osteopontin

## Abstract

**Background:**

Osteopontin (OPN) is a secreted phosphoprotein expressed by neoplastic cells involved in the malignant potential and aggressive phenotypes of human malignancies, including gastrointestinal stromal tumors (GISTs). Our previous study showed that OPN can promote tumor cell proliferation in GISTs. In this series, we further aim to investigate the effect of OPN on apoptosis in GISTs.

**Methods:**

The expression of apoptotic and anti-apoptotic proteins in response to OPN was evaluated. *In vitro* effects of OPN against apoptosis in GIST were also assessed. GIST specimens were also used for analyzing protein expression of specific apoptosis-related molecules and their clinicopathologic significance.

**Results:**

Up-regulation of β-catenin and anti-apoptotic proteins Mcl-1 with concomitant suppression of apoptotic proteins in response to OPN was noted. A significant anti-apoptotic effect of OPN on imatinib-induced apoptosis was identified. Furthermore, Mcl-1 overexpression was significantly associated with OPN and β-catenin expression in tumor tissues, as well as worse survival clinically.

**Conclusions:**

Our study identifies anti-apoptotic effects of OPN that, through β-catenin-mediated Mcl-1 up-regulation, significantly antagonized imatinib-induced apoptosis in GISTs. These results provide a potential rationale for therapeutic strategies targeting both OPN and Mcl-1 of the same anti-apoptotic signaling pathway, which may account for resistance to imatinib in GISTs.

## Background

Gastrointestinal stromal tumors (GISTs), the most common type of gastrointestinal tract mesenchymal tumor, originate from the interstitial cells of Cajal in the myenteric plexus of the digestive tract, with the stomach being the most common tumor location. The pathogenesis of GIST is gain-of-function mutations of the proto-oncogene *KIT* in the majority, or platelet-derived growth factor receptor α (PDGFRA), with resultant encoding of related proteins, KIT, or PDGFRA receptors that contain ligand-independent kinase activity, leading to persistent and uncontrolled cell proliferation as well as resistance to apoptosis [1,2]. It has also recently been proposed that ETV1, one of the *ETS* family transcription factors, cooperates with KIT in the tumorigenesis of GIST [3].

Surgery remains the standard and curative treatment of choice for patients with a resectable GIST. Target therapy using imatinib mesylate, a KIT receptor inhibitor, is indicated in those with advanced or unresectable GIST, or in high-risk patients after surgery as an adjuvant therapy [4,5]. However, acquired or secondary resistance to imatinib may occur in GIST patients under imatinib treatment for disease recurrence or progression [6,7]. It is therefore important to identify the mechanisms underlying imatinib-resistance so that therapeutic interventions can be developed and applied to this particular GIST patient group.

Osteopontin (OPN), initially termed Eta-1, standing for early T cell activation gene 1, or spp1, abbreviated from secreted phosphoprotein 1, was originally identified as a secreted protein from transformed mammalian cells [8,9]. OPN is currently known as a multifunctional secreted glycophosphoprotein expressed in many cell and tissue types and has been found to participate in numerous cellular functions of both physiologic and pathologic significance [10,11]. OPN, being frequently overexpressed in miscellaneous tumor cell types, plays important roles in their malignant potential and aggressiveness, including tumor growth, invasion, metastasis, survival, angiogenesis, and tumorigenesis [12-14].

The clinical significance of OPN as a biomarker for poor prognosis has been reported in many human malignancies, including GISTs [12,15-18]. In addition to the clinicopathologic significance of OPN that independently predicts poor clinical outcomes in GIST, we also identified that OPN, upon its interaction with CD44, a type I transmembrane receptor that recognizes OPN as one of its important ligands, contributes to tumor cell proliferation in GIST cell lines [9,19]. These data add to evidence supporting the role of OPN as well as its interaction with CD44 in the functional regulation of cancer cells [17,18].

In addition to increased proliferation, anti-apoptosis or suppression of apoptosis, is a common strategy tumor cells use for tumor progression and drug-resistance. Based on the significance of OPN in the antagonistic regulation of apoptosis noted in normal cells as well as in malignant cells [20-24], pharmaceutical inhibition of OPN as the target for induction of apoptosis has been proposed in several human malignancies experimentally and clinically [25,26]. Since tumor cell proliferation and apoptosis are closely related and usually coexisting hallmark features characteristic of cancer cells, including GIST, and since we have identified the effect of OPN on promoting tumor proliferation in GIST, we then further hypothesize that OPN may also play a role in the regulation of apoptosis in GIST. This study therefore aims to investigate the effects of OPN in relation to apoptosis and anti-apoptosis in GIST.

## Methods

### Cell lines and cell culture

GIST cell lines, including imatinib-sensitive GIST882, and imatinib-resistant GIST48B and GIST62, were kindly provided by Dr. Chen from the National Health Research Institute (Tainan, Taiwan) and were maintained in RPMI media (Invitrogen) with 10% fetal bovine serum (Sigma) in a humidified atmosphere with 5% CO_2_ and 95% air at 37°C.

### Construction of shRNA and transient transfection

The shRNA against OPN was conducted by delivery of shRNA plasmid DNA into cells using the lipofectamine method with a ratio of 5 μg DNA per 30 μL lipofectamine (Qiagen, Hilden, Germany). Cells transfected with plasmid of luciferase gene serve as the vector control. After DNA transfection, cells are grown in regular medium for 48 hours and transfection efficacy is evaluated by luciferase bioactivity.

### Patients and tissue samples

Between January 2002 and January 2012, 31 patients undergoing surgery for primary GIST at the National Cheng Kung University Hospital were included for evaluation. The diagnosis of GIST was confirmed by positive KIT immunohistochemical staining of tumor specimens. The surgical principle for each patient was to achieve complete tumor excision with microscopically negative margins. Patients received regular follow-up at the outpatient department for abdominal ultrasonography or CT every three months. No patients received adjuvant or neoadjuvant therapy. Written informed consent was obtained from all patients and the institute review board of the National Cheng Kung University Hospital approved this study.

### Western blotting analysis

GIST cell lines GIST882, GIST48B, and GIST62 as well as tumor tissues preserved in liquid nitrogen (-80°C) from 31 GIST patients were used for western blotting analysis. Pair-matched normal tissues from 16 of these 31 patients were also used for comparison. Tumor specimens were lysed with PBS/TDS lysis buffer as described previously [17,18]. The tissue lysate was centrifuged at 15,000 × *g* for removal of insoluble cellular materials (Beckman Coulter). Protein concentrations were measured by the Pierce BCA protein assay (Pierce). Equal amounts of protein were subjected to electrophoresis on 10% sodium dodecyl sulfate-polyacrylamide gel electrophoresis gels (Amersham Pharmacia Biotech) and then transferred to PVDF filters (Perkin-Elmer). GIST882 cell lysate samples of 30 μg were used for the same procedures. The filters were then blocked in PBS containing 10% skim milk (BD) for 60 minutes and subsequently incubated with a 1:500 dilution of rabbit polyclonal anti-CD44 antibody (Abcam; ab65829), a 1:50 dilution of mouse monoclonal anti-β-catenin antibody (Santa Cruz; sc7963), a 1:1,000 dilution of rabbit polyclonal anti-Mcl-1 antibody (Santa Cruz; sc-819), rabbit monoclonal anti-Bak antibody (Abcam; ab32371), rabbit polyclonal anti-XIAP antibody (Abcam; ab2541), and a 1:10,000 dilution of mouse monoclonal anti-β-actin antibody (Sigma; A1978) in PBS containing 0.03% Tween 20 overnight. The PDVF filters were then washed three times for 5 minutes with PBS containing 0.3% Tween 20 (Merck) and then incubated for 60 minutes with horseradish peroxidase-conjugated anti-rabbit IgG antibody (Amersham) for anti-CD44cyto antibody, anti-Mcl-1 antibody, anti-Bak antibody, anti-XIAP antibody, and with horseradish peroxidase-conjugated anti-mouse IgG (GE) for anti-β-catenin antibody and anti-β-actin antibody. Detection of secondary antibodies was done by using an enhanced chemiluminescence system (Amersham). We used the muscle tissue of the paired-match normal tissue as the normal control of beta-catenin in Western blot analysis. The positive control of the western blot analysis of Mcl-1 in GIST was the HEK293 cell lysate.

### Terminal deoxynucleotidyl transferase-mediated dUTP nick end labeling (TUNEL) assay

Equal amounts of GIST882 cells were deposited on glass slides for fixation. TUNEL was performed using TACS-2 TdT-Fluor *In Situ* Apoptosis Detection Kit (Trevigen) according to the manufacturer’s instructions. The apoptotic index (per × 200 microscopic field) was calculated as the number of apoptotic cells × 200 per total number of cells under a fluorescence microscope (Olympus, Tokyo, Japan).

### Immunohistochemistry

Formalin-fixed and paraffin-embedded blocks from 31 GIST tumor specimens were prepared and treated as previously described [27]. The same protocol was used for detection of immunoreactivity, counterstaining, and selection of positive and negative controls. The primary antibodies used were mouse monoclonal anti-OPN antibody (Santa Cruz; sc21742) at 1:500 dilution and rabbit polyclonal anti-Mcl-1 antibody (Santa Cruz; sc-819) at 1:1,000 dilution. The secondary antibodies and avidin-biotin-complex were then applied. Semiquantitative evaluation of the immunohistochemical staining was done by two investigators blinded to the clinical information separately. The grading of OPN expression was categorized according to the same criteria as described previously [18].

### Statistical analysis

Comparison between the two groups was done by independent sample *t*-test for continuous variables and χ^2^ test was employed for analysis of categorical variables. Significant variables in the univariate analysis were subjected to a multivariate logistic regression for determination of the independent significance of specific parameters. One-way ANOVA was used for comparisons of the means of more than two groups. Correlations between continuous variables were analyzed by Pearson’s correlation coefficients. Survival curves were measured by the Kaplan-Meier method. Disease-specific survival was defined as the survival period between the diagnosis of the disease and the death of the patient due to disease recurrence after surgery. Log-rank test was used for comparison of survival between groups. Statistical analyses were performed using the SPSS computer statistical software (SPSS software; version 13.0; Chicago, IL, USA). A *P* value of <0.05 was considered significant in each analysis.

## Results

### Expression of apoptotic and anti-apoptotic proteins in response to OPN in GIST cell lines

**Figure 1:**
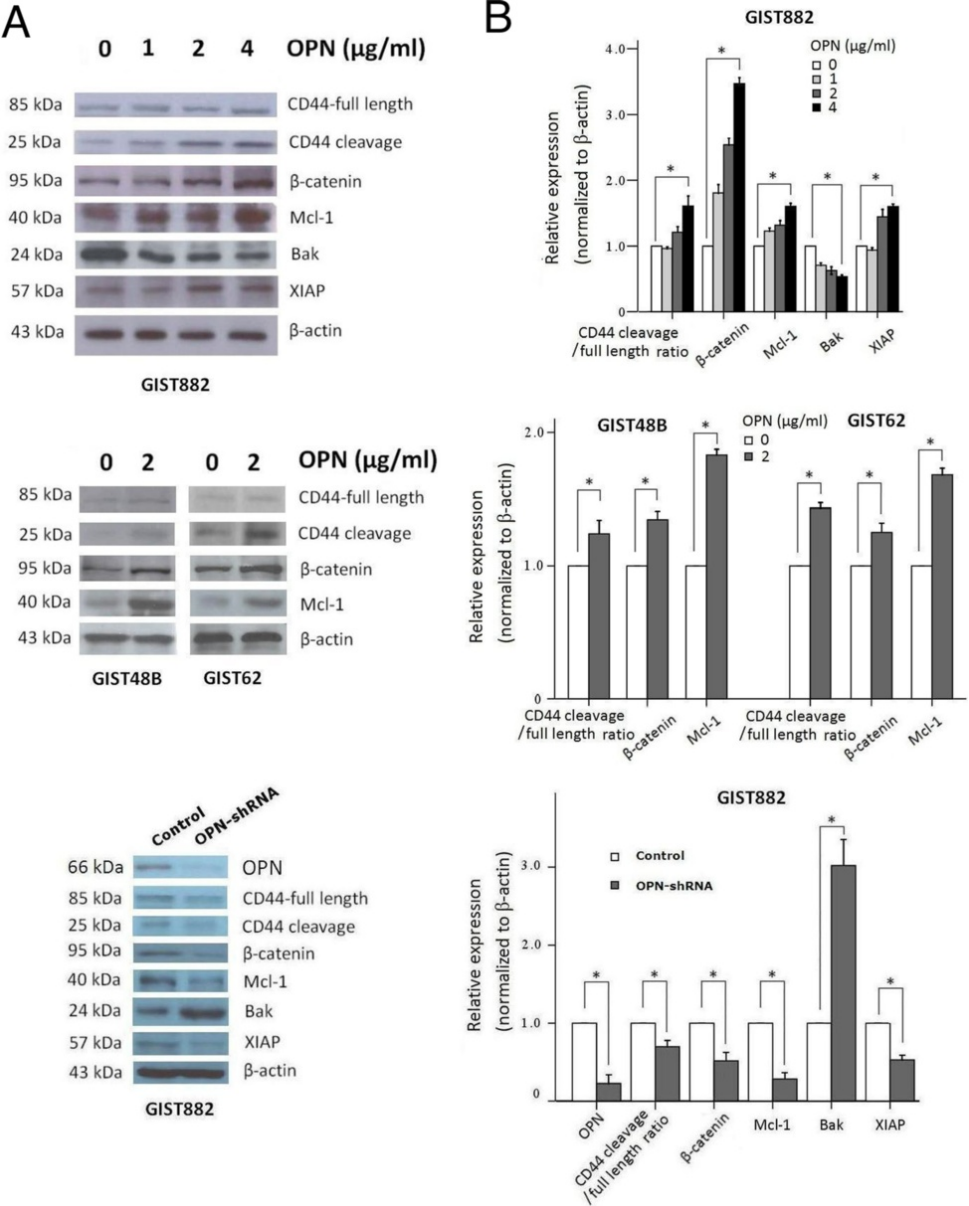
**Assessment of the activities of anti-apoptotic and apoptotic proteins in response to OPN in GIST. (A)** Western blotting analysis of the total protein extracts from GIST882, GIST48B, and GIST62 cell lysates. Knockdown assessment of OPN by siRNA in GIST882 cell line was also demonstrated in the lower panel. **(B)** Quantitation of the western blots by using β-actin as the control, demonstrating increased activities of the anti-apoptotic proteins, Mcl-1 and XIAP, with concomitant suppressed activities of apoptotic proteins Bak, after OPN administration in a dose-dependent manner in imatinib-sensitive GIST882. There were also increased activities of CD44 cleavage and β-catenin (upper). Similar effects of OPN in promoting the expression of β-catenin and Mcl-1 were also noted in imatinib-resistant GIST48B and GIST62 (Columns, mean of results in triplicate; bars, SD. *, *P* <0.05).

To assess the *in vitro* expression of apoptotic and anti-apoptotic proteins in GIST, we treated the GIST882, sensitive to imatinib, and imatinib-resistant GIST48B and GIST62 GIST cell lines with OPN at different concentrations and analyzed the protein expression of specific apoptosis-related proteins by western blotting. In the anti-apoptotic proteins analyzed, we found increased expression of Mcl-1 and XIAP with correspondingly decreased expression of the apoptotic protein Bak, in response to OPN administration dose-dependently in GIST882. Knockdown of OPN in GIST882 cell lines showed inhibition of the anti-apoptotic effects of OPN demonstrated above (Figure 1). These data suggest a potential *in vitro* anti-apoptotic effect of OPN via up-regulation of anti-apoptotic proteins in GIST882. Similar effects of OPN in promoting the expression of β-catenin and Mcl-1 were also noted in imatinib-resistant GIST48B and GIST62. In our previous studies, we identified that OPN may interact with CD44 to induce CD44 cleavage, possibly through increased expression of both β-catenin and cyclin D1 in GIST tumor tissues [17]. Western blotting using GIST882, GIST48B, and GIST62 cell lines in this study demonstrated compatible results. Dose-dependent induction of CD44 cleavage and β-catenin expression in GIST cell lines upon OPN administration was observed regardless of drug-resistance (Figure 1).

### *In vitro* anti-apoptotic effects of OPN on imatinib-induced apoptosis in GIST

**Figure 2:**
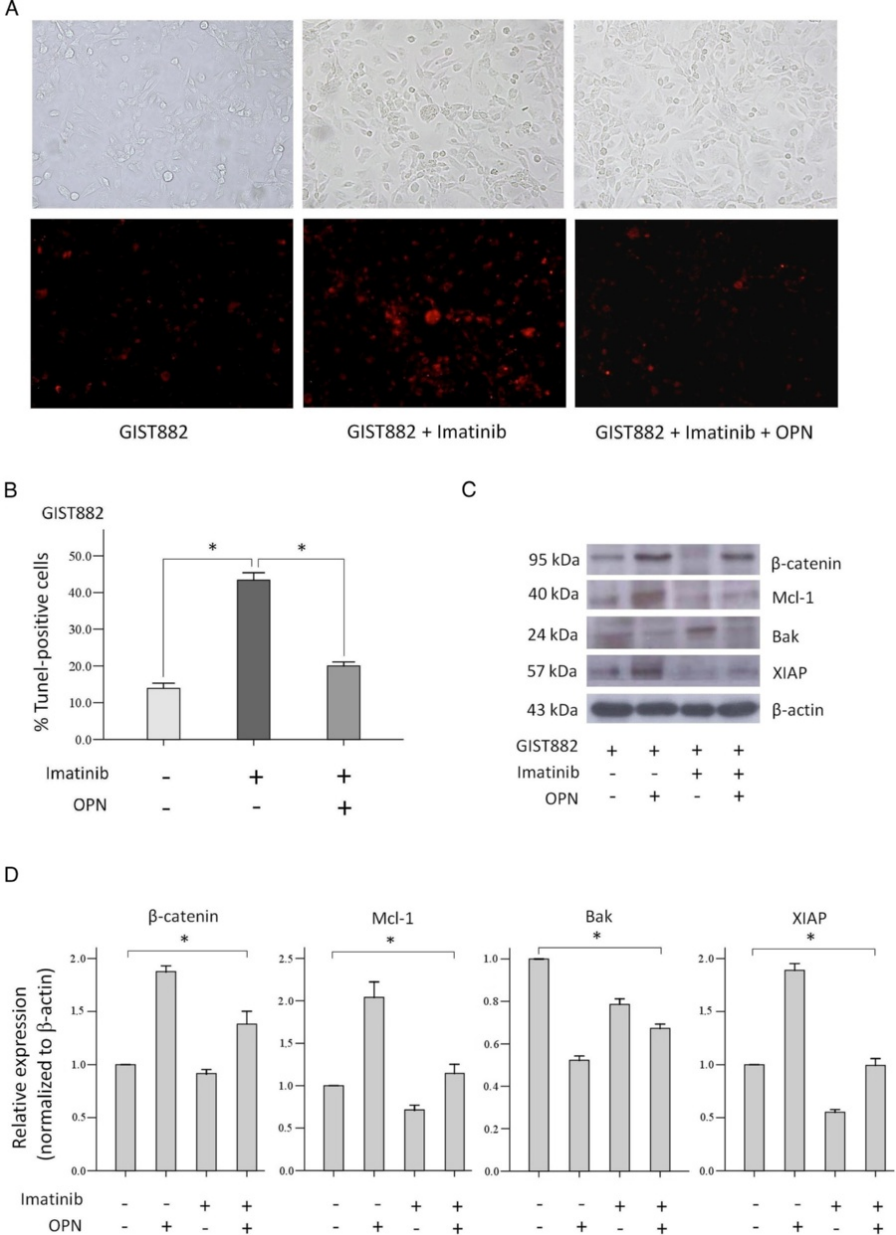
**Antagonistic effect of OPN on imatinib-induced apoptosis in GIST882 by TUNEL assay. (A)** Representative images indicating changes in the number of TUNEL-positive GIST882 tumor cells treated with imatinib (1 μM) with or without OPN administration (2 μg/mL). (Original magnification: ×200). **(B)** The number of TUNEL-positive tumor cells increased significantly in GIST882 treated with imatinib, whereas their numbers decreased significantly with simultaneous administration of OPN upon imatinib treatment, indicating an *in vitro* anti-apoptotic effect of OPN in GIST (Columns, mean of results in triplicate; bars, SD. *, *P* <0.001) . **(C)** Western blotting analysis of GIST882 cell lines treated with imatinib and/or OPN, corresponding to the treatment used in the TUNEL assay. **(D)** Quantification of the western blots indicating that significant changes in the expression of anti-apoptotic and apoptotic proteins, in response to OPN in GIST882 cell lines treated with imatinib, were similar to changes in the percentage of apoptotic cells in the TUNEL assay (Columns, mean of results in triplicate; bars, SD. *, *P* <0.05, one-way ANOVA).

**Figure 3:**
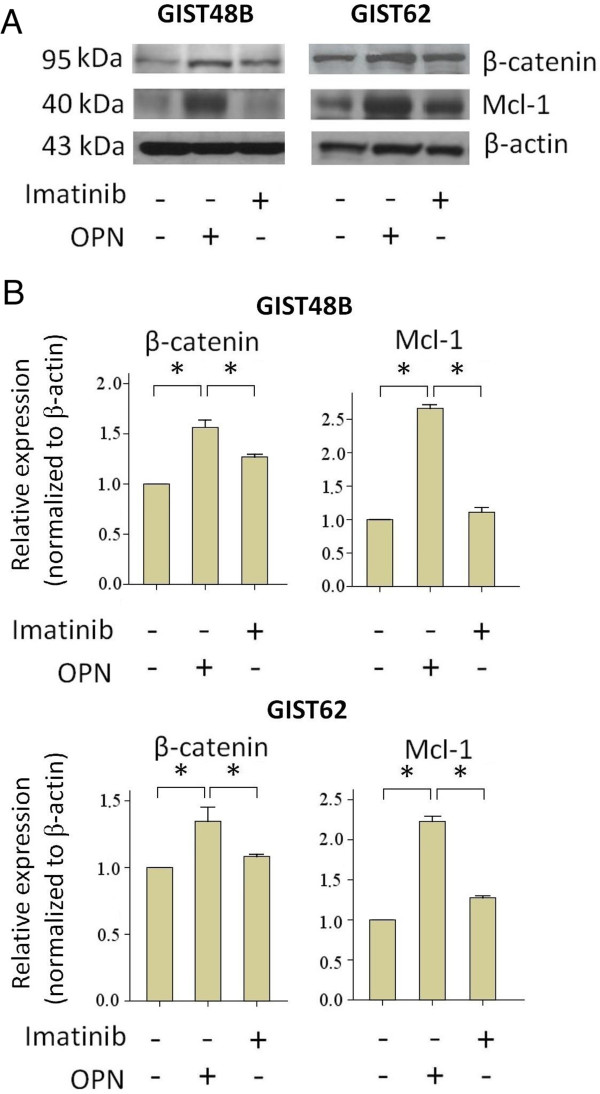
**Upregulation of β-catenin and anti-apoptotic protein Mcl-1 in response to OPN in GIST cell lines resistant to imatinib. (A)** Western blotting analysis of GIST48B and GIST62 cell lines treated with imatinib (1 μM) or OPN (2 μg/mL). **(B)** Quantification of the western blots showing significant changes in the expression of β-catenin and anti-apoptotic protein Mcl-1 in relation to OPN administration in GIST48B and GIST62 (Columns, mean of results in triplicate; bars, SD. *, *P* <0.05).

Imatinib is an inhibitor of both normal and mutated KIT found in most GISTs and has been used for the treatment of patients with advanced GIST or for adjuvant therapy after surgical resection [4,5]. Imatinib has also been widely used to induce apoptosis in GIST882. We therefore used imatinib to further evaluate the anti-apoptotic effects of OPN in GIST882, the known imatinib-sensitive GIST cell line [28]. In the TUNEL assay for apoptosis analysis, GIST882 exhibited significant tumor cell apoptosis upon imatinib treatment in the absence of OPN, as expected. However, this pro-apoptotic effect of imatinib on GIST882 was significantly inhibited, or antagonized, upon OPN administration (Figure 2A,B), indicating an *in vitro* anti-apoptotic effect of OPN in GIST. We also analyzed the expression of apoptotic and anti-apoptotic proteins by western blotting in the three groups of GIST882 cell lines used in the TUNEL assay and found that significant changes in the expression level of β-catenin, Mcl-1, and XIAP were compatible with and corresponding to the results from the TUNEL assay (Figure 2C,D). Similarly, in imatinib-resistant GIST cell lines GIST48B and GIST62, significant upregulation of β-catenin and anti-apoptotic protein Mcl-1 in response to OPN was noted (Figure 3). Taken together, these findings suggest a significant anti-apoptotic effect of OPN against imatinib-induced apoptosis in GIST.

In conclusion, we suggest that OPN contributes to significant anti-apoptotic effects in GIST, against imatinib-related apoptosis specifically, through the mechanism of up-regulation of β-catenin and anti-apoptotic protein Mcl-1, with concomitant suppression of apoptotic proteins.

### Significant correlation of β-catenin and anti-apoptotic protein Mcl-1 expression in GIST

**Figure 4:**
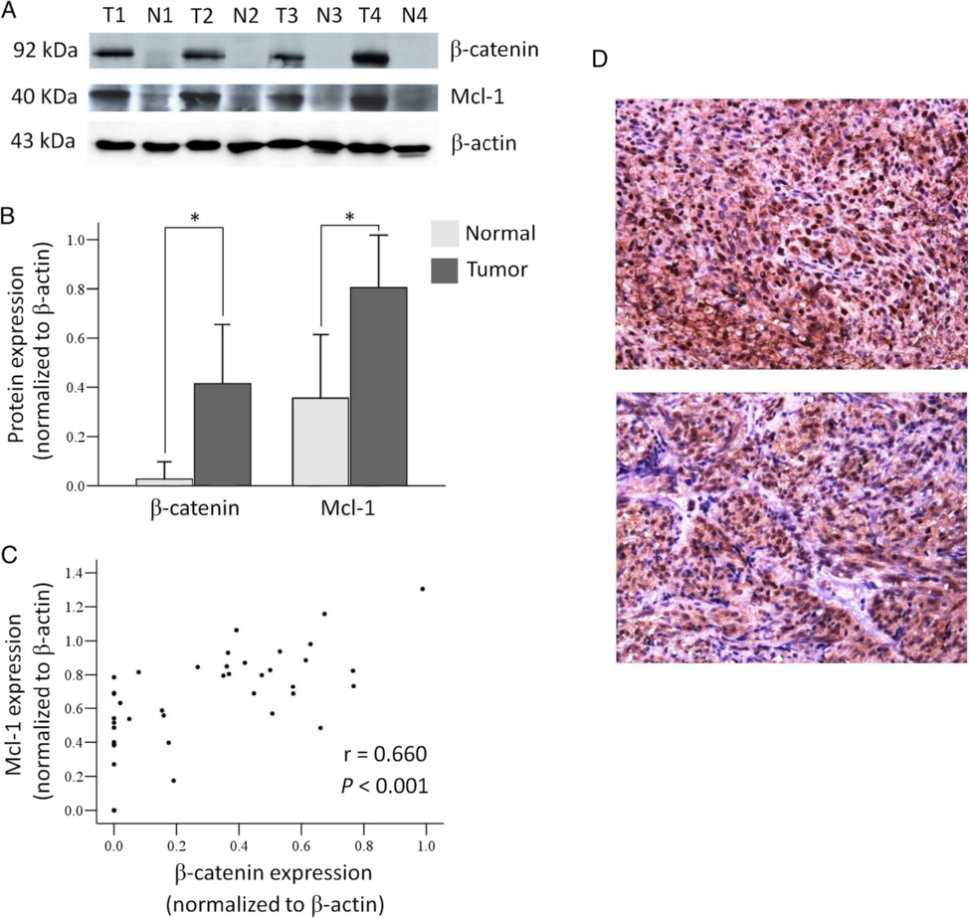
**Expression and correlation of β-catenin and Mcl-1 in GIST. (A)** Representative tumor (T) and normal (N) tissue samples analyzed by immunoblotting with anti-β-catenin, anti-Mcl-1, and anti-β-actin antibodies. **(B)** Comparison of the average β-catenin and Mcl-1 protein expression by western blotting analysis in tumor tissues (mean ± SD, 0.42 ± 0.25 and 0.80 ± 0.21, respectively, n = 31) and normal counterpart tissues (mean ± SD, 0.03 ± 0.07 and 0.36 ± 0.26, respectively, n = 16). Columns, mean; bar, SD. *, *P* <0.001. Equal amounts of protein loading were ensured using β-actin as the internal control. **(C)** There was a significantly positive correlation between β-catenin and Mcl-1 expression by Pearson correlation analysis (r = 0.660; *P* <0.001). **(D)** Representative immunohistochemical staining showing strong immunoreactivity for OPN (upper) and Mcl-1 (lower) in GIST. Original magnification, ×200.

In our previous investigations of the interaction of OPN and CD44 and the associated clinicopathologic significances in GIST, β-catenin was found likely to play an important role in the regulation of downstream signaling and functional effects subsequent to OPN and CD44 interaction [17]. In accordance with other series suggesting that OPN may be related to the induction of β-catenin or Wnt signaling pathways for regulating tumor progression, including resistance to apoptosis, in cancer cell lines [26,29-31], we confirmed that β-catenin can be a downstream effecter of OPN in GIST and may be regulating the expression of anti-apoptotic protein Mcl-1 (Figures 1– 3). To further evaluate the association of β-catenin and Mcl-1, we analyzed the expression of both proteins in 31 patients with GIST by western blotting in the tumor specimens and normal counterpart tissues (Figure 4A). Having been normalized to β-actin for quantification of protein expression, both β-catenin and Mcl-1 exhibited tumor-specific overexpression, with significantly increased level of expression in tumor tissues relative to normal tissues (*P* <0.001). The average protein expression levels of β-catenin and Mcl-1 were 0.42 ± 0.25 and 0.80 ± 0.21 (mean ± SD), respectively (Figure 4B). Additionally, we also analyzed the correlation between the expression levels of β-catenin and Mcl-1 and found that these two proteins showed significant positive correlations (Figure 4C). These results further support the close association between β-catenin and Mcl-1, as well as the significant regulatory role of β-catenin in GIST, as demonstrated in the western blotting analysis in GIST cell lines (Figures 1– 3).

### Clinicopathologic significance of Mcl-1 expression in GIST

**Figure 5:**
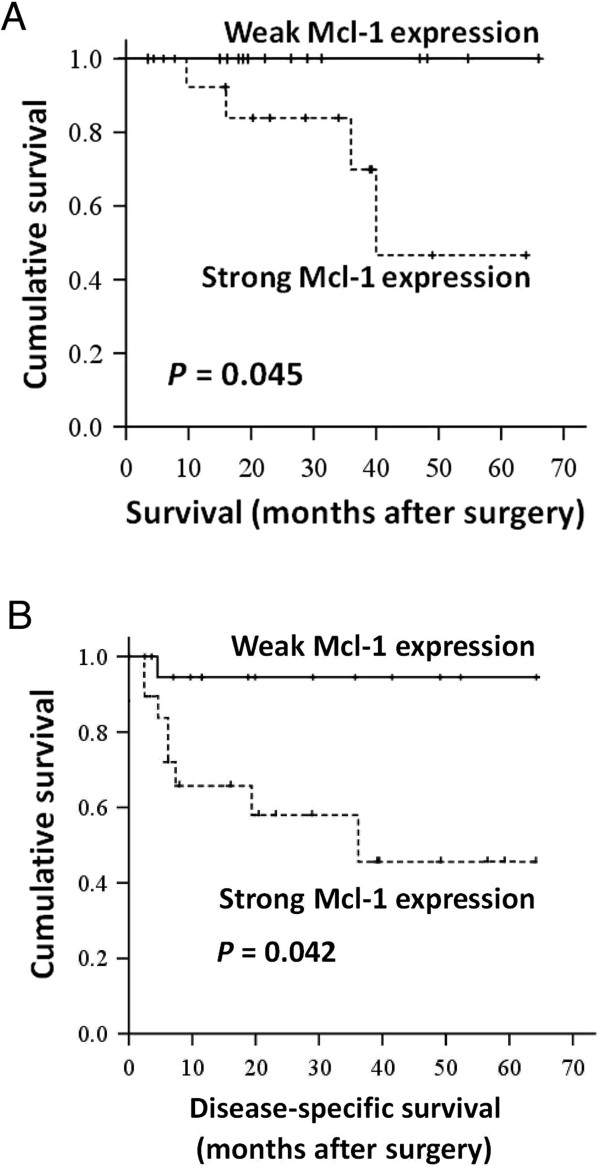
**Kaplan-Meier survival curves for overall survival (A) and disease-specific survival (B) in patients with GIST with respect to Mcl-1 expression.** Log-rank test reveals that patients with strong Mcl-1 expression were significantly associated with worse overall and disease-specific survivals. The *P* value of the log-rank test is also shown.

In GIST tumor specimens, increased expression of OPN and Mcl-1 can also be observed by immunohistochemical staining (Figure 4D). The expression and grading of OPN in GIST has been previously described [17]. To further evaluate the clinicopathologic significance of Mcl-1 expression in GIST, we use the mean tumor Mcl-1 expression value of 0.8 by western blotting as the cut-off level to define Mcl-1 activity in patients with GIST. Patients with Mcl-1 levels ≥0.8 were those with strong Mcl-1 expression and others were those with weak Mcl-1 expression. Table 1 demonstrated the demographics and clinicopathological data in 31 patients with GIST with strong or weak Mcl-1 expression. No difference existed between the two groups in terms of age, gender, symptoms and signs, tumor size, mitotic figures, and recurrence. However, patients with strong Mcl-1 expression were significantly associated with a higher proportion of tumor localization at the stomach (*P* = 0.020) and strong OPN expression (*P* = 0.007). Subsequent multivariate analysis of these parameters revealed that only strong OPN expression remained the independent factor associated with increased Mcl-1 expression, indicating a significant and close correlation between OPN and Mcl-1 in GIST. These clinicopathologic data may also partly provide additional support to our previous *in vitro* findings that OPN contributes to significant anti-apoptotic effects in GIST through up-regulation of the anti-apoptotic protein Mcl-1. In the Kaplan-Meier estimation for overall and disease-specific survivals, patients with strong Mcl-1 expression showed worse overall and disease-specific survivals, compared with those with weak Mcl-1 expression (*P* = 0.045 and 0.042, respectively) (Figure 5). These results revealed a prognostic value of Mcl-1 expression in patients with GIST.

**Table 1:** Demographics and clinicopathological data in patients with GIST according to Mcl-1 expression

	**Mcl-1 expression**				
	**Weak**	**Strong**	**Total No. of patients (%)**	**Univariate analysis**	**Multivariate analysis**
	**<0.8**	**≥0.8**			
	**No. of patients (%)**	**No. of patients (%)**		***P *** value ^*^	***P *** value ^*^
Number of patients	15 (48.4)	16 (51.6)	31 (100)	–	–
Age (years), mean ± SD	57.3 **±** 14.4	63.2 **±** 11.8	60.4 ± 13.3	0.225	0.505
Sex					
Male	7 (46.7)	8 (50)	15 (48.4)	0.853	0.465
Female	8 (53.3)	8 (50)	16 (51.6)		
Symptom and signs					
Yes	12 (80)	11 (68.7)	23 (74.2)	0.474	
No	3 (20)	5 (31.3)	8 (25.8)		
Tumor location					
Stomach	5 (33.3)	12 (75)	17 (54.8)	0.020	0.051
Intestine	10 (66.7)	4 (25)	14 (45.2)		
Tumor size (cm), mean ± SD	8.0 ± 5.2	7.4 ± 4.5	7.7 ± 4.8	0.745	
Mitosis (HPF), mean ± SD	20.3 ± 22.9	16.1 ± 31.7	18.1 ± 27.4	0.672	
Recurrence					
Yes	5 (33.3)	4 (25)	9 (29)	0.609	
No	10 (66.7)	12 (75)	22 (71)		
Osteopontin expression^**^					
Strong	5 (33.3)	13 (81.3)	18 (58.1)	0.007	0.015
Weak	10 (66.7)	3 (18.7)	13 (41.9)		

SD indicates standard deviation; HPF: high-power fields.

^*^Significant at the level of *P* <0.05.

^**^Strong: 3+; Weak: <3 + .

## Discussion

The anti-apoptotic role of OPN has been described in several human malignancies, including hepatocellular carcinoma (HCC), colorectal cancer, pancreatic cancer, breast cancer, lung cancer, prostate cancer, and glioma [22-24]. In this study, we identified the anti-apoptotic effect of OPN in GIST for the first time. This anti-apoptotic effect of OPN, through β-catenin-mediated up-regulation of anti-apoptotic protein Mcl-1, was able to attenuate imatinib-induced apoptosis in GIST *in vitro*, suggesting a possible role of OPN and Mcl-1 in the mechanism underlying drug resistance to imatinib in GIST patients. The results from our study were similar to those of one report in which OPN was found to up-regulate the anti-apoptotic protein Bcl-2 to cause inhibition of apoptosis, thus enhancing resistance to chemotherapy in lung cancer cell lines, indicating the involvement of OPN in the development of acquired chemoresistance [16]. One report also proposed that OPN, being a main anti-apoptotic factor that inhibited caspase 3-dependent apoptosis, was responsible for resistance to chemotherapy in a murine breast cancer cell line model [32]. Another study also identified that enhanced multidrug resistance in mesothelioma cells was induced and mediated through the anti-apoptotic effect of OPN [33]. In line with these studies, our finding that OPN exhibited significant anti-apoptotic effects in GIST, and specifically in the presence of imatinib, through anti-apoptotic protein Mcl-1 up-regulation, may be of value in supporting the rationale for development of new pharmaceutical strategies against imatinib-resistant GIST, which may be common in patients with disease recurrence or progression after imatinib treatment [6,7]. Our previous study has proved OPN overexpression to be associated with tumor recurrence in patients with resectable GIST [18]. Further studies are necessary to investigate the role of OPN expression in the particular GIST patient group undergoing neoadjuvant or adjuvant imatinib treatment that subsequently developed drug-resistance.

Some studies have focused on therapeutic agents or manipulations targeting apoptosis-related molecules in the treatment of GIST. Pharmaceutical molecules used to induce or promote apoptosis in GIST include Bortezomib, a dipeptide boronic acid inhibitor of the 20S proteasome that, in part, impedes the degradation of pro-apoptotic factors, thereby inducing apoptosis in neoplastic cells, and MegaFaL, a synthetic compound containing a hexameric form of soluble forms of Fas ligand (FasL; CD95L), which binds to Fas receptor for induction of Fas-mediated apoptosis [34,35]. One series also proposed that ABT-737, the pro-apoptotic Bcl-2/Bcl-xL inhibitor, can synergistically promote imatinib-induced cytotoxicity via apoptosis [36]. Our study suggests that OPN, with its igniting or initiating role of specific anti-apoptotic signaling transduction via up-regulation of anti-apoptotic protein Mcl-1 and its significant anti-apoptotic effect on inhibiting imatinib-induced apoptosis, may be a potential and promising target for pharmaceutical intervention in GIST resistant to conventional imatinib treatment. Along with our previous findings [17,18], we identified the biologic significance of OPN in GIST to be associated with dual modes of actions, including proliferation-promoting as well as anti-apoptotic effects, indicating a pivotal role of OPN in tumor progression and malignant potential in GIST. These also provide evidence supporting OPN as an important target of pharmaceutical intervention in the treatment of GIST.

Mcl-1, a member of the anti-apoptotic Bcl-2 family, was originally cloned as an immediate-early induction gene expressed during differentiation of the ML-1 myeloid leukemia cells and functions as a regulator for survival and development in diverse cell types physiologically. In tumor biology, Mcl-1 plays an oncogenic role by maintaining tumor cell survival through its anti-apoptotic function [37,38]. Overexpression of Mcl-1 has been reported in a wide variety of human cancers, including gastrointestinal malignancies such as colorectal cancer, gastric cancer, and HCC [39-41]. Our study is the first to document Mcl-1 overexpression, its association with OPN anti-apoptotic effects, and its prognostic significance in predicting poor survival in GIST. We also identified a positive correlation in the expression of β-catenin and Mcl-1 in both GIST tumor specimens and GIST cell lines, suggesting that β-catenin may be an effector of OPN-mediated up-regulation of Mcl-1 and anti-apoptosis in GIST. However, to further verify and conclude this putative mechanism of OPN-mediated up-regulation of Mcl-1 through β-catenin in GIST proposed in our series, subsequent functional studies may be necessary. A similar regulatory role of OPN in β-catenin functions for anti-apoptosis has also been proposed in another study in which OPN was found to promote β-catenin signaling to induce resistance to apoptosis in prostate cancer cells [24].

It has been reported that Mcl-1 promotes cell survival by suppressing cytochrome c release from mitochondria through neutralization of pro-apoptotic proteins including Bak [42]. This is compatible with our finding that increased Mcl-1 expression was coupled with deceased expression of Bak, which may be part of the signaling pathway accounting for the anti-apoptotic function of Mcl-1 in response to OPN in GIST (Figure 1).

Mcl-1 has also been implicated as a key element in the resistance to conventional cancer therapies in certain human malignancies, including chronic myeloid leukemia, HCC, and cholangiocarcinoma [43-46]. The significance of Mcl-1 in its association with drug resistance and its overexpression in these human malignancies makes Mcl-1 a potential therapeutic target for intervention and treatment. However, though Mcl-1 overexpression in GIST was related to poor survival in this study, we have also noted the lack of association of strong Mcl-1 expression with mitotic rate, tumor size, and tumor location, three widely recognized and strongly validated prognostic parameters of GISTs. As revealed by the title of the study, the major finding of this study is the anti-apoptotic effects of OPN through up-regulation of Mcl-1 in GIST. Mcl-1, being an anti-apoptotic protein, may be more likely to be an important mediator and participant in the signal pathways involved in the anti-apoptotic effects of OPN in GIST, rather than the different mechanisms regarding tumor proliferation and progression. It is therefore reasonable that the expression of the anti-apoptotic protein Mcl-1, is not directly associated with mitotic rate and tumor size, two major reliable indicators and poor prognostic factors for tumor proliferation, growth, and progression in GIST. According to our study, OPN, through up-regulation of anti-apoptotic protein Mcl-1, antagonized imatinib-induced apoptosis in GIST *in vitro*, suggesting a potential role of OPN and Mcl-1 in the mechanism underlying drug resistance to imatinib in GIST. As a result, Mcl-1 is more likely to be involved in the anti-apoptosis and drug resistance in GIST. This potential role of Mcl-1 in GIST drug-resistance merits further investigations. Our study also interestingly identified that strong Mcl-1 expression was associated with the favorable prognostic factor of gastric location in the univariate analysis, although this was only of borderline significance in the multivariate analysis (*P* = 0.051); the significance of this association is not clear. However, this is similar to another study in which CD133, being more significantly and more frequently expressed in gastric GIST (48%) than in intestinal GIST (4%), was also found to be a significant poor prognostic factor regarding overall survival in GIST [47]. It is notable that CD133, one of the well-recognized cancer stem cell markers in the GI tract, may potentially be involved in drug-resistance in GIST. This is also similar to the proposed role of Mcl-1 in the anti-apoptosis and drug-resistance in GIST in our study and awaits further investigation.

In this study, we found a significant association of OPN and increased Mcl-1 expression (Figure 1 and Table 1). We thus propose that OPN and Mcl-1 may be an upstream initiator and downstream effector, respectively, of the same anti-apoptotic signal pathway, in their contribution to resistance to imatinib-induced apoptosis in GIST. It is theoretically possible to simultaneously inhibit or target these two anti-apoptotic molecules on the same axis of survival signaling in order to obtain synergistic efficacy for induction of GIST tumor cell apoptosis. Interestingly, recent studies have identified that cyclooxygenase-2 inhibitors can induce apoptosis and anti-tumor activities through down-regulation of OPN as well as Mcl-1 [29,48,49]. We therefore consider the use of cyclooxygenase-2 inhibitors for induction of apoptosis via down-regulation of dual targets of OPN and Mcl-1 as a promising strategy in the treatment of GIST, especially in imatinib-resistant GIST; further *in vitro* and *in vivo* studies are undergoing.

## Conclusion

In conclusion, we identified the anti-apoptotic effect of OPN in GIST for the first time. This anti-apoptotic effect of OPN, through β-catenin-mediated up-regulation of anti-apoptotic protein Mcl-1, also antagonized imatinib-induced apoptosis in GIST *in vitro*, suggesting a potential role of OPN and Mcl-1 in the mechanism underlying drug resistance to imatinib in GIST patients. Our results therefore provide evidence supporting the rationale for therapeutic strategies targeting both OPN and Mcl-1 to interrupt associated anti-apoptotic signaling in drug-resistant GIST for prevention of tumor progression.

## Abbreviations

GIST: Gastrointestinal stromal tumor; HCC: Hepatocellular carcinoma; OPN: Osteopontin; TUNEL: Terminal deoxynucleotidyl transferase-mediated dUTP nick end labeling.

## Competing interests

The authors declare that they have no competing interests.

## Authors’ contributions

KHH carried out the conception and design of the study as well as acquisition and interpretation of the data. HWT and YSH contributed to the experiment and analyzed the data. PWL, PJL, and YSS made critical review of the manuscript. All authors read and approved the final version of the manuscript.
